# Periodontal Outcomes in Anterior Teeth following Presurgical Orthodontic Decompensation in Patients with Skeletal Class III Malocclusion: A Single-Arm Systematic Review and Meta-Analysis

**DOI:** 10.1155/2024/5020873

**Published:** 2024-08-24

**Authors:** Yun He, Siyuan Wang, Hui Xiong

**Affiliations:** ^1^ Hospital of Stomatology Wuhan University, Wuhan, Hubei Province, China; ^2^ Department of Orthodontics Hospital of Stomatology Wuhan University, Wuhan, Hubei Province, China

## Abstract

**Objective:**

This study aims to systematically review and analyze the periodontal outcomes of presurgical orthodontic decompensation (POD) in patients with skeletal Class III malocclusion and to identify the key influencing factors. *Material and Methods*. We searched the Web of Science, PubMed, Scopus, Embase, and Cochrane Library databases. The outcomes included measurements related to periodontal soft or hard tissues.

**Results:**

A total of 3,904 records were found, of which 10 were included. The meta-analysis revealed significant alveolar bone loss in mandibular incisors on both the lingual and labial sides during POD, with a more pronounced loss on the lingual side at the apex level and on the labial side near the crown. The maxillary incisors demonstrated significant bone loss, primarily on the lingual side. No significant bone loss was observed during postsurgical orthodontic treatment. Gingival recession was statistically significant but had a minor clinical impact. Incisor proclination was found to influence the gingival recession. However, no correlation was observed between bone loss and incisor proclination, vertical facial type, or sex.

**Conclusions:**

POD for skeletal Class III patients results in alveolar bone loss, particularly on the lingual side at the mandibular incisors' apex level and labial side at the crown level, and clinically acceptable gingival recession.

## 1. Introduction

Class III malocclusion is characterized by mandibular prognathism, maxillary hypoplasia, or a combination of both [1]. Surgical orthodontic treatment typically involves presurgical orthodontic decompensation (POD), orthognathic surgery to correct skeletal discrepancies, and orthodontic finishing.

Prolonged misalignment before treatment often results in compensated occlusion, characterized by proclined maxillary and retroclined mandibular incisors in patients with Class III malocclusion. POD aims to retrocline the maxillary incisors and procline the mandibular ones. Although orthodontic treatment can enhance bone healing [2], improper force distribution may lead to bone defects. Some studies have shown that POD benefits root position and bone condition in incisors [3]. However, other studies have found no correlation between periodontal recession and labial movement [4, 5, 6], while some have found it to result in bone defects [7, 8, 9], particularly in patients with Class III malocclusion [10]. These findings affect esthetics and oral health, highlighting the need for periodontal monitoring during POD. In some cases, a surgery-first orthognathic approach is employed to minimize the labial movement of the mandibular incisors and avoid bone defects [11]. However, its effectiveness remains controversial [11, 12].

Given these varying findings, it is crucial to review and synthesize previous research to inform future studies and clinical practice. This systematic review and meta-analysis aimed to analyze periodontal changes during POD in the anterior region.

## 2. Materials and Methods

### 2.1. Registration

This study was registered in PROSPERO (Registration ID: CRD42023480753) and was conducted according to the PRISMA statement [13].

### 2.2. Eligibility Criteria

We employed the PICOS criteria for study inclusion:

Population: Patients diagnosed with skeletal Class III malocclusion, aged 15–40 years, were included if they were free from severe periodontal diseases, severe crowding or facial asymmetry, cleft lip/palate, severe craniofacial syndromes, or systemic diseases.

Intervention: Patients underwent both orthodontic decompensation treatment and orthognathic surgery.

Study design: Experimental and observational studies were included, excluding case reports.

Outcome: We included studies reporting at least one parameter related to periodontal hard or soft tissues.

### 2.3. Searching Process

We retrieved studies from the following five databases: PubMed, Cochrane Library, Scopus, Embase, and Web of Science. The search included publications from the earliest available date up to July 5, 2024. Additionally, we screened the references in the selected studies.

Two reviewers (Yun He and Siyuan Wang) independently screened the searched studies. In cases of disagreement, an arbitrator, Dr. Hui Xiong, was consulted to reach a consensus. The search strategy is detailed in *Supplementary table 1*.

### 2.4. Data Collection

Two researchers independently extracted data, encompassing basic information, such as sample size, participant characteristics, age, sex distribution, ethnicity, intervention, treatment duration, indicators of incisor inclination, and outcome indicators related to soft or hard tissues.

### 2.5. Risk of Bias and Quality Assessment

Risk of bias assessment of the included studies was independently performed by two reviewers. The Newcastle–Ottawa scale (NOS) was used for quality assessment of the cohort studies, while the JBI critical appraisal checklist for case series [14] was used to evaluate the quality of the case series. Detailed questions regarding the checklist are presented in *Supplementary table 2*.

The quality of the included studies was assessed by two reviewers using the Grading of Recommendations Assessment, Development, and Evaluation (GRADE) approach [15].

### 2.6. Summary of Measurements and Synthesis of Results

The meta-analysis was performed using Review Manager 5.4 (Nordic Cochrane Center, Cochrane Collaboration, Copenhagen, Denmark). Continuous data were presented as mean ± SD or transformed accordingly. The summary effect measure used was the weighted mean difference and its corresponding 95% confidence interval (CI). Statistical heterogeneity was assessed using the *χ*^2^ and *I*^2^ tests. A random effects model was used. Statistical significance was set at *p*  < 0.05. Methods and outcomes that could not be quantitatively analyzed were qualitatively described.

### 2.7. Reporting Bias Assessment and Sensitivity Analysis

As a single-arm rate meta-analysis, we report only descriptive results without the presence of “positive” or statistically significant findings. Therefore, no reporting bias assessment or sensitivity analysis was performed in this study.

## 3. Results

### 3.1. Study Selection

Following our search strategy, 792 records were identified in PubMed, 82 in the Cochrane Library, 1,575 in Scopus, 1,086 in Embase, and 906 in Web of Science. After removing 537 duplicate records, the remaining total was 3,904 records. No study meeting the inclusion criteria was found in the electronic search of “gray” literature.

Two reviewers (Yun He and Siyuan Wang) independently screened the titles and abstracts, excluding 3,815 studies. Among these, 1,052 were excluded because they did not meet the population inclusion criteria, 998 for intervention noncompliance, 854 for not meeting the study design criteria, and 911 for not meeting the outcome criteria. Subsequently, 89 studies underwent full-text evaluation, and ultimately, 10 studies were included. The references in these studies were reviewed, and no further studies were included.

**Figure 1:**
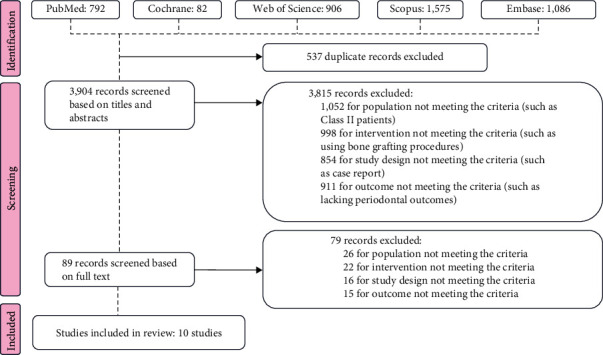
Flow diagram of searching process.

The screening process is shown in Figure 1.

### 3.2. Study Characteristics

We identified four cohort studies [11, 16, 17, 18] and five case series [3, 19, 20, 21, 22, 23]. All the studies were published as full-text articles in English, except for one published in Chinese [3]. Regarding funding, two studies did not receive any funding [18, 23], six received funding from universities and the government [3, 16, 17, 19, 20], and one from a dental association [22]. One study did not report its funding resource [21].

The synthesized participant age was 22.15 (SD = 2.44), with one study not reporting participant ages [16]. The synthesized female ratio of the participants was 51.61%. All patients were diagnosed with Class III malocclusion with proclined maxillary incisors and retroclined mandibular incisors. Additionally, all of them underwent orthognathic and orthodontic treatments. All studies investigated the mandibular central incisor, and several included the mandibular lateral incisor [3, 17, 18, 21, 22] or the maxillary central incisor [19, 20, 21, 23]. Patients with severe dental crowding, facial asymmetry, cleft lip and palate, or facial deformities were excluded.

The detailed study characteristics are presented in Table 1. The interventions for each study and the patients' facial characteristics are presented in Table 2.

### 3.3. Risk of Bias and Quality Assessment

The results of the risk of bias assessment are presented in Table 3. According to the NOS, three cohort studies [11, 17, 18] were rated as good quality, while one study [16] was rated as fair quality. The case series were evaluated using the JBI critical appraisal checklist for case series. One study [19] was rated as good quality, three [3, 20, 22] as fair quality, and two [21, 23] as poor quality. The results of the poor-quality studies were not synthesized.

The quality assessment of the mandibular central incisors demonstrated low certainty, mainly because of the nonrandomized study design. Only the outcome of the mandibular central incisors before orthognathic surgery was evaluated because studies on other tooth sites were relatively rare, and the outcome of the orthodontic treatment after surgery did not show statistical significance.

### 3.4. Synthesized Measurements of the Alveolar Bone

**Figure 2:**
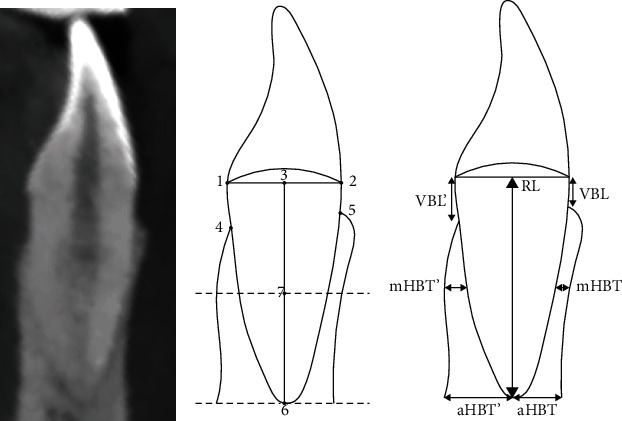
Illustration of alveolar bone measurements. (a) A CBCT section; (b and c) an illustrative diagram. 1, cementoenamel junction on lingual side; 2, cementoenamel junction on labial side; 3, midpoint of points 1 and 2; 4, alveolar ridge crest on lingual side; 5, alveolar ridge crest on labial side; 6, root apex; 7, midpoint of points 3 and 6; VBL, distance between points 2 and 5; VBL', distance between points 1 and 4; RL, distance between points 3 and 7; mHBT and mHBT', respective bone thickness measured perpendicular to RL and through point 7 on labial side and on lingual side; aHBT and aHBT', respective bone thickness measured perpendicular to RL and through point 6 on labial side and on lingual side; %VBL, calculated by VBL/root × 100%; and %VBL', calculated by VBL'/root × 100%.

Nine studies [3, 11, 16, 18, 19, 20, 21, 22, 23] reported the outcome of the alveolar bone for the mandibular central incisor; four studies [3, 18, 21, 22] reported for the mandibular lateral incisor; and three studies [19, 20, 23] reported for the maxillary central incisor. Among them, five reported that the typical measurements that could be synthesized [11, 16, 19, 20, 22], including vertical alveolar bone level on the labial side (VBL) and on the lingual side (VBL'), ratio of VBL to root length on the labial side (VBL%) and on the lingual side (VBL%'), horizontal bone thickness at midroot level on the labial side (mHBT) and on the lingual side (mHBT'), and horizontal bone thickness at apex on the labial side (aHBT) and on the lingual side (aHBT'). The detailed measurement standards are shown in Figure 2.

Statistically significant bone loss was observed in the incisors from before POD (T0) to after treatment (T1). The mandibular central incisor was commonly investigated (*n* = 117), and bone loss was severe on the labial side near the crown in a vertical direction, with a VBL increase of 1.33 mm (95% CI = [0.90, 1.76]), VBL% increase of 12.30% (95% CI = [7.89, 18.10]), mHBT decrease of 0.21 mm (95% CI = [−0.02, 0.44]), and aHBT increase of 0.81 mm (95% CI = [0.01, 1.62]). Severe bone loss was also reported on the lingual side near the apex region, with a VBL increase of 2.20 mm (95% CI = [1.17, 3.23]), VBL% increase of 16.84% (95% CI = [5.25, 28.44]), mHBT decrease of 0.50 mm (95% CI = [0.33, 0.67]), and aHBT decrease of 1.54 mm (95% CI = [1.05, 2.04]). Maxillary incisors also exhibited statistically significant and predominant bone loss, primarily on the lingual side.

During the orthognathic surgery and postsurgery orthodontic treatment (from T1 to T2), the alveolar bone condition was stable, and no bone loss was observed.

**Figure 3:**
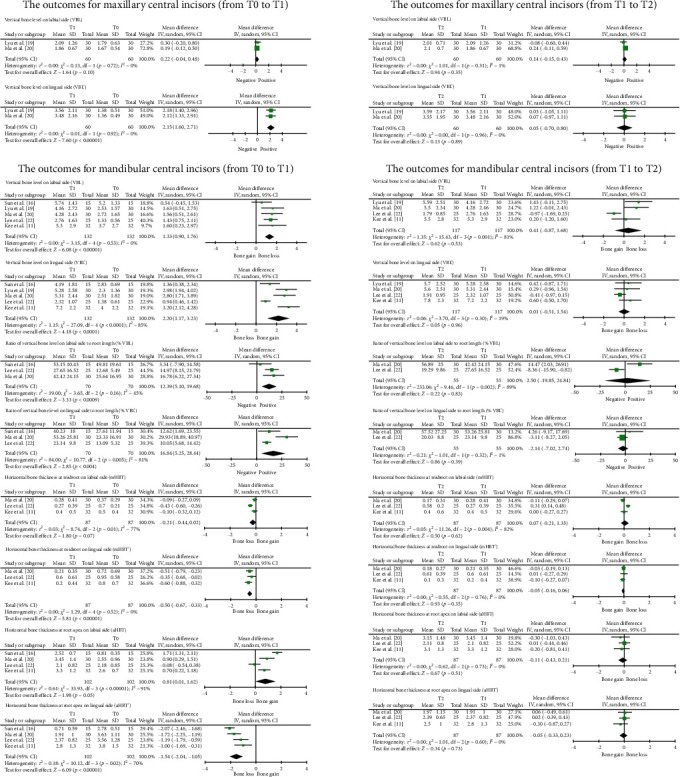
Forest plot of all alveolar bone measurements. All measured distances are in millimeters (mm).

The detailed outcomes of the synthesized bone conditions are summarized in Table 4, and a forest plot is shown in Figure 3.

### 3.5. Synthesized Measurement of Periodontal Soft Tissue

Choi et al. [18] reported the T0 and T1 conditions in the periodontal soft tissue of the anterior mandibular teeth. In their experimental group, the free gingival margin on the labial side exhibited a mean vertical recession of 0.37 mm (SD = 0.91), and the mean width of the attached gingiva decreased by 0.75 mm (SD = 1.09). The differences in these two indicators before and after the orthodontic treatment, as well as the differences in the changes between the experimental and control groups, all reached statistical significance, though they were clinically insignificant.

### 3.6. Influencing Factors

Potential factors influencing changes in periodontal tissues mentioned in the literature include patient sex, age, incisor inclination, vertical facial type, treatment duration, gingival biotypes, and so on.

No statistically significant correlation was observed between periodontal condition and sex (two studies [18, 21]), nor was there a correlation observed between alveolar bone loss in the mandibular anterior teeth and patient vertical facial type [17].

In studies by Sun et al. [16], Kurt et al. [21], and Lee et al. [22], no statistically significant correlation was observed between changes in lower incisor inclination and bone remodeling quantity. However, Choi et al. [18] reported that the proclination of the lower central incisor significantly affected the width of the attached gingiva. More specifically, the width of the attached gingiva decreased as the tip of the mandibular incisor moved forward.

## 4. Discussion

Our study found that POD for patients with skeletal Class III malocclusion leads to periodontal bone loss and soft tissue recession in the incisors. This contrasts with some studies suggesting that it would contribute to bone gain [3].

For the mandibular incisors, bone loss was observed on both the labial and lingual sides. Bone loss was severe on the lingual side, both vertically and horizontally, at both the mid-root and root apex levels. Conversely, bone loss occurred vertically near the crown on the labial side. It was less significant near the mid-root level and even showed bone gain at the apex level. Regarding the maxillary incisors, the synthesized results suggested significant bone defects on the lingual side of the alveolar bone during POD, in contrast to the findings in the mandibular incisors. Kim et al. [23] compared the difference between the mandible and maxilla and found that the mandibular alveolar bone underwent more bone loss during POD and had a worse condition after POD, especially at the lingual side at the root apex level. This is consistent with the synthesized data in this review.

Bone loss can be attributed to a change in the position of the incisors. In patients with compensated skeletal Class III mandibular incisors, the lingual alveolar bone is inherently thin. POD retroclines the teeth, potentially causing the roots to protrude through the cortical bone, leading to fenestration and dehiscence. In studies reporting alveolar bone gain [3], precise root control may have contributed to this outcome. By proclining and retruding the incisor, root position was adjusted from an unfavorable position before treatment, potentially preventing bone resorption and aiding bone regeneration [3]. Therefore, root control should be the primary focus during POD. Techniques such as CBCT should be used to determine alveolar bone limitation, which may reduce alveolar bone loss and even improve bone condition.

Some studies have attributed bone loss to excessive orthodontic force, leading to microfractures [24]. However, it is worth noting that this has a smaller impact on POD. Previous research has demonstrated that adopting the surgical-first approach, which avoids excessive orthodontic force, does not result in less alveolar bone loss [11, 12].

As for alveolar bone loss from T1 to T2, no bone loss was detected during the postsurgical orthodontic treatment. Some studies [22] reported that postsurgical bone loss was not significant, whereas others [19, 20] reported otherwise. Other factors, such as postoperative inflammation and the postoperative relapse, may have contributed to this variation.

Studies on soft tissue were limited. Although, Kurt et al. [21] demonstrated a statistically significant gingival recession during POD, the amount observed was less than 1 mm, which falls within clinically acceptable limits, thereby sufficiently preserving periodontal conditions [25].

### 4.1. Influencing Factors

The magnitude of incisor proclination is a critical factor that may influence the periodontal condition. Although three existing studies [16, 21, 22] did not find a significant correlation between changes in incisor inclination and alveolar bone defects, Choi et al. [18] observed a significant impact on attached gingiva.

Yao et al. [17] reported no statistically significant correlation between patients vertical facial type and alveolar bone loss in the anterior mandibular teeth. However, several studies [17] have demonstrated that patients with a high preoperative angle tend to have poorer alveolar bone conditions. Ahn et al. [26] indicated that the vertical facial type of patients could affect the presurgical orthodontic treatment of the mandibular incisors in Class III malocclusion. Considering the relatively small sample size in Yao et al.'s study [17] and the potential influence of other factors, further research is warranted.

### 4.2. Limitation

This study has certain limitations, including confounding the results of cohort studies with those of case series, variations in measuring indicators across different studies, leading to the inability to synthesize certain indicators, differences in orthodontic treatment protocols, inclusion of only high-angle patients in some studies, inclusion of patients with all vertical facial types in others, and a limited sample size. The scarcity of research on soft tissue conditions is also a notable limitation, and future researchers may further investigate this aspect.

The findings of this review serve as a valuable reference for understanding periodontal changes following POD, aiding orthodontists in treatment planning and predicting outcomes. For patients with Class III malocclusion and poor alveolar bone conditions, additional treatment such as periodontally accelerated osteogenic orthodontics (PAOO) may be considered.

## 5. Conclusion

POD for skeletal Class III malocclusion can result in alveolar bone loss and acceptable gingival recession in the mandibular incisors.

For mandibular incisors, greater alveolar bone loss is evident on the lingual side, with the condition of the labial side bone also being critical, particularly near the crown.

For the maxillary incisors, statistically significant alveolar bone loss was observed on the lingual side but not on the labial side.

## Figures and Tables

**Table 1:** Characteristics of the included studies.

Characteristics	Lee et al. [22]	Ma et al. [20]	Yao et al. [17]	Sun et al. [16]	Lyu et al. [19]	Demirsoy et al. [21]	Choi et al. [18]	Kim et al. [23]	Zhao et al. [3]	Kee et al. [11]
Design	Case series	Case series	Cohort	Cohort	Case series	Case series	Cohort	Case series	Case series	Cohort

Country	South Korea	China	China	China	China	Turkey	Korea	Korea	China	South Korea

Public language	English	English	English	English	English	Engish	English	English	Chinese	English

Participants (*n*)	25	30	29	15	30	26	39	20	15	32

Age	26.3 ± 2.7	20.94 ± 3.25	21.2 ± 3.7	>18	20.53 ± 2.86	17.44 ± 2.80	22.1 ± 4.2	24.1 ± 4.2	25.5 ± 3.6	23.5 ± 6.2

Female (%)	48	50	44.8	NR	50	53.8	61.5	55	40	53.1

Tooth site	Mandibular central and lateral incisors	Mandibular and maxillary central incisors	Mandibular central incisors, lateral incisors, and canine	Mandibular central incisors	Mandibular and maxillary central incisors	Mandibular and maxillary central and lateral incisors	Mandibular central and lateral incisors	Mandibular and maxillary central incisors	Mandibular central incisors, lateral incisors, and canine	Mandibular central incisors

Periodontal outcomes	Alveolar bone height and thickness	Alveolar bone height and thickness	Alveolar bone height and thickness	Alveolar bone height and thickness	Alveolar bone height and thickness	Bone dehiscence and fenestration	Sulcus probing depth, bone probing depth, and width of the attached gingiva	Alveolar bone height and thickness	Bone dehiscence and fenestration and root movement	Alveolar bone height, thickness, and area

Risk of bias assessment	Fair quality	Fair quality	Good quality	Fair quality	Good quality	Poor quality^a^	Good quality	Poor quality^a^	Fair quality	Good quality

^a^Result was not synthesized due to poor quality but involved in subsidiary analysis.

**Table 2:** The intervention for each research study and patients' facial characteristics.

Intervention		Lee et al. [22]	Ma et al. [20]	Yao et al. [17]	Sun et al. [16]	Lyu et al. [19]	Demirsoy et al. [21]	Choi et al. [18]	Kim et al. [23]	Zhao et al. [3]	Kee et al. [11]
Orthodontic appliance		Straight wire fixed appliance	Straight wire fixed appliance	Straight wire fixed appliance	Straight wire fixed appliance	Straight wire fixed appliance and anchorage in the maxillary arch	Straight wire fixed appliance	NR	NR	Straight wire fixed appliance	NR

Tooth extraction		NR	Bilateral maxillary first premolar extracted	NR	10 patients had teeth extracted,5 did not	Bilateral maxillary first premolar extracted	NR	NR	NR	NR	NR

Decompensation achieved	U1-SN	NR	T0 = 108.52 ± 7.27 T1 = 105.48 ± 5.44 T2 = 106.13 ± 7.34	NR	NR	NR	T0 = 107.35 ± 7.29 T1 = 104.96 ± 7.19	NR	NR	NR	T0 = 109.8 ± 6.3 T1 = 108.8 ± 6.2 T2 = 110.8 ± 6.5
	IMPA	T0 = 82.63 ± 7.94 T1 = 92.17 ± 6.43 T2 = 87.42 ± 7.31	T0 = 75.75 ± 8.68 T1 = 85.86 ± 7.37 T2 = 84.37 ± 7.32	T0 (low angle) = 81.54 ± 5.08 T0 (normal angle) = 79.39 ± 5.95 T0 (high angle) = 76.03 ± 6.45	T0 = 78.79 ± 6.08 T1 = 90.12 ± 4.14	NR	T0 = 76.91 ± 5.54 T1 = 85.01 ± 5.84	D = 14.18 ± 3.57	T1 = 86.6 ± 8.7	D = −1.20 ± 0.10	T0 = 82.4 ± 6.3 T1 = 87.0 ± 6.2 T2 = 84.8 ± 6.0

Surgical correction done	SNA	T0 = 80.57 ± 3.44 T1 = 80.58 ± 3.28 T2 = 81.71 ± 2.63	T0 = 77.48 ± 3.21 T1 = 77.21 ± 2.9 T2 = 80.97 ± 3.80	NR	T0 = 80.81 ± 3.30 T1 = 80.81 ± 3.30	NR	T0 = 78.66 ± 3.58 T1 = 78.62 ± 3.58	T0 = 81.1 ± 3.3	T1 = 81.8 ± 4.4	NR	T0 = 80.6 ± 2.7 T1 = 80.5 ± 2.7 T2 = 80.6 ± 2.8
	SNB	T0 = 83.41 ± 2.42 T1 = 84.13 ± 3.81 T2 = 79.69 ± 3.10	T0 = 82.13 ± 3.23 T1 = 82.12 ± 3.53 T2 = 79.45 ± 3.60	NR	T0 = 84.41 ± 4.12 T1 = 84.93 ± 4.05	NR	T0 = 83.09 ± 3.78 T1 = 83.13 ± 3.74	T0 = 84.2 ± 4.2	T1 = 85.4 ± 5.4	NR	T0 = 83.4 ± 3.2 T1 = 82.8 ± 3.0 T2 = 79.9 ± 2.9
	ANB	T0 = 22.45 ± 2.44 T1 = 22.75 ± 2.89 T2 = 2.03 ± 2.74	T0 = 4.67 ± 2.49 T1 = 4.89 ± 2.50 T2 = 1.51 ± 1.89	T0 (low angle) = −10.76 ± 2.48 T0 (normal angle) = − 12.49 ± 5.79 T0 (high angle) = −11.86 ± 4.84	T0 = −4.63 ± 1.89 T1 = −4.82 ± 1.93	NR	T0 = −4.43 ± 2.40 T1 = −4.50 ± 2.41	NR	T1 = −3.6 ± 2.6	NR	T0 = 109.8 ± 6.3 T1 = 108.8 ± 6.2 T2 = 110.8 ± 6.5

NR, not reported.

**Table 3:** Risk of bias assessment of the studies included in the meta-analysis.

Study	Q1	Q2	Q3	Q4	Q5	Q6	Q7	Q8	Q9	Q10	Overall
JBI critical appraisal checklist for case series for the included retrospective studies											
Lee et al.[22]	Yes	Yes	Yes	Unclear	Unclear	Yes	Yes	Yes	No	Yes	Fair
Ma et al. [20]	Yes	Yes	Yes	No	Unclear	Yes	Yes	Yes	No	Yes	Fair
Lyu et al. [19]	Yes	Yes	Yes	Yes	Unclear	Yes	Yes	Yes	No	Yes	Good
Demirsoy et al. [21]	Yes	Yes	Yes	No	Unclear	Yes	Yes	Yes	No	No	Poor
Kim et al. [23]	Yes	Yes	Yes	No	Unclear	Yes	Yes	No	No	Yes	Fair
Zhao et al. [3]	Yes	Yes	Yes	Unclear	Unclear	Yes	Yes	Yes	No	Yes	Fair

Study	I	II	III	IV	V	VI	VII	VIII			Overall

Newcastle–Ottawa scale (NOS) for the included nonrandomized studies											
Yao et al. [17]	1	1	1	1	1	1	1	1			Good
Sun et al. [16]	0	0	1	1	1	1	0	1			Fair
Choi et al. [18]	1	1	1	1	1	1	1	1			Good
Kee et al. [11]	1	1	1	1	1	1	1	1			Good

**Table 4:** Summary of synthesized outcomes for alveolar bone measurement.

Tooth site	Outcome	Changes for presurgical orthodontic treatment			Changes for postsurgical orthodontic treatment		
		Mean	95% CI	*p*	Mean	95% CI	*p*
Mandibular central incisors	VBL	1.33	[0.90, 1.76]	<0.00001 ^*∗∗∗*^	0.41	[−0.87, 1.68]	0.53
	VBL'	2.20	[1.17, 3.23]	<0.00001 ^*∗∗∗*^	0.01	[−0.51, 0.54]	0.96
	VBL%	12.39	[7.89, 18.10]	0.0009 ^*∗∗∗*^	2.50	[−19.85, 24.84]	0.83
	VBL%'	16.84	[5.25, 28.44]	0.004 ^*∗∗*^	−2.14	[−7.02, 2.74]	0.39
	mHBT	−0.21	[−0.44, 0.02]	0.07	0.07	[−0.21, 0.35]	0.62
	mHBT'	−0.50	[−0.67, −0.33]	<0.0001 ^*∗∗∗*^	−0.05	[−0.16, 0.06]	0.35
	aHBT	0.81	[0.01,1.62]	0.05 ^*∗*^	−0.11	[−0.33, 0.23]	0.73
	aHBT'	−1.54	[−2.04, −1.05]	<0.00001 ^*∗∗∗*^	−0.05	[−0.43, 0.21]	0.51

Mandibular lateral incisors	VBL	1.58	0.71–2.45	0.0004 ^*∗∗∗*^	−0.81	−1.72–0.10	0.08
	VBL'	0.98	0.28–1.68	0.006 ^*∗∗*^	−0.78	−1.47- (−0.09)	0.03 ^*∗*^
	VBL%	15.83	7.82–23.84	0.0001 ^*∗∗∗*^	−7.15	−15.47–1.17	0.09
	VBL%'	8.41	1.16–15.66	0.02 ^*∗*^	−6.62	−13.88–0.64	0.07
	mHBT	−0.28	−0.40- (−0.16)	<0.0001 ^*∗∗∗*^	0.12	0.00–0.24	0.05 ^*∗*^
	mHBT'	−0.25	−0.60–0.10	0.16	0.11	−0.23–0.45	0.53
	aHBT	−0.14	−0.48–0.20	0.42	0.26	−0.14–0.66	0.21
	aHBT'	−1.21	−1.84- (−0.58)	0.0002 ^*∗∗∗*^	−0.04	−0.62–0.54	0.89

Maxillary central incisors	VBL	0.22	[−0.04, 0.48]	0.10	0.14	[−0.15, 0.43]	0.35
	VBL'	2.15	[1.6, 2.71]	<0.00001 ^*∗∗∗*^	0.05	[−0.70, 0.80]	0.89
	VBL%	3.92	[0.54, 7.30]	0.02 ^*∗*^	3.32	[−1.21, 7.85]	0.15
	VBL%'	23.53	[14.76, 32.30]	<0.00001 ^*∗∗∗*^	1.87	[−9.82, 13.56]	0.75
	mHBT	0.12	[−0.05, 0.29]	0.18	−0.27	[−0.49, −0.05]	0.01 ^*∗∗*^
	mHBT'	−1.44	[−1.86, −1.02]	<0.00001 ^*∗∗∗*^	−0.12	[−0.54, 0.30]	0.58
	aHBT	0.61	[0.06, 1.16]	0.03 ^*∗*^	0.10	[−0.57, 0.77]	0.77
	aHBT'	−1.29	[−2.15, −0.43]	0.003 ^*∗∗*^	−0.74	[−1.67, 0.19]	0.12

All measured distances are in millimeters (mm). CI, confidence interval;  ^*∗*^, *p* ≤ 0.05;  ^*∗∗*^, *p* ≤ 0.01;  ^*∗∗∗*^, *p* ≤ 0.001.

## Data Availability

Data sharing is not applicable to this article as it is a review article.

## Supplementary Materials

Supplementary 1 Table 1: detailed searching strategy for different databases.

Supplementary 2 Table 2: questions from the JBI critical appraisal checklist and the Newcastle–Ottawa scale (NOS) used for risk of bias assessment.
