# Supplementary material for: Periodontal Outcomes in Anterior Teeth following Presurgical Orthodontic Decompensation in Patients with Skeletal Class III Malocclusion: A Single-Arm Systematic Review and Meta-Analysis
Source: Int J Dent. 2024 Aug 24;2024:5020873. doi: 10.1155/2024/5020873 (PMC11366050; doi:10.1155/2024/5020873)
Supplement: Supplementary 1 — Table 1: detailed searching strategy for different databases. [file 5020873.f1.docx]

Searching strategy for different data base

| **Data base** | **Searching strategy** |
| --- | --- |
| **Pubmed** | ("orthodontics"[MeSH Terms] OR "orthodontics"[Title/Abstract] OR "orthodontic"[Title/Abstract]) AND ("presurgical"[Title/Abstract] OR "orthognathic"[Title/Abstract] OR "before surgery"[Title/Abstract] OR "decompensation"[Title/Abstract]) AND ("bone"[Title/Abstract] OR "periodontal"[Title/Abstract] OR "soft tissue"[Title/Abstract]) |
| **Web Of Science** | TS=("orthodontic") AND (TS=("presurgical") OR TS=("orthognathic") OR TS=("before surgery") OR TS=("decompensation")) AND (TS=("bone") OR TS=("periodontal") OR TS=("soft tissue")) |
| **Cochrane Library** | (MH "Orthodontics" OR orthodontics:ti,ab,kw OR orthodontic:ti,ab,kw) AND (presurgical:ti,ab,kw OR orthognathic:ti,ab,kw OR "before surgery":ti,ab,kw OR decompensation:ti,ab,kw) AND (bone:ti,ab,kw OR periodontal:ti,ab,kw OR "soft tissue":ti,ab,kw) |
| **Scopus** | (TITLE-ABS-KEY("orthodontics") OR TITLE-ABS-KEY("orthodontic")) AND (TITLE-ABS-KEY("presurgical") OR TITLE-ABS-KEY("orthognathic") OR TITLE-ABS-KEY("before surgery") OR TITLE-ABS-KEY("decompensation")) AND (TITLE-ABS-KEY("bone") OR TITLE-ABS-KEY("periodontal") OR TITLE-ABS-KEY("soft tissue")) |
| **Embase** | ('orthodontics'/exp OR 'orthodontics':ti,ab OR 'orthodontic':ti,ab) AND ('presurgical':ti,ab OR 'orthognathic':ti,ab OR 'before surgery':ti,ab OR 'decompensation':ti,ab) AND ('bone':ti,ab OR 'periodontal':ti,ab OR 'soft tissue':ti,ab) |
