# Supplementary material for: Periodontal Outcomes in Anterior Teeth following Presurgical Orthodontic Decompensation in Patients with Skeletal Class III Malocclusion: A Single-Arm Systematic Review and Meta-Analysis
Source: Int J Dent. 2024 Aug 24;2024:5020873. doi: 10.1155/2024/5020873 (PMC11366050; doi:10.1155/2024/5020873)
Supplement: Supplementary 2 — Table 2: questions from the JBI critical appraisal checklist and the Newcastle–Ottawa scale (NOS) used for risk of bias assessment. [file 5020873.f2.docx]

**Questions in JBI Critical Appraisal Checklist and the Newcastle–Ottawa Scale (NOS)**

JBI Critical Appraisal Checklist for case Series for included retrospective studies：

Q1, were there clear criteria for inclusion in the case series?

Q2, was the condition measured in a standard, reliable way for all participants included in the case series?

Q3, were valid methods used for identification of the condition for all participants included in the case series?

Q4, did the case series have consecutive inclusion of participants?

Q5, did the case series have complete inclusion of participants?

Q6, was there clear reporting of the demographics of the participants in the study?

Q7, was there clear reporting of clinical information of the participants?

Q8, were the outcomes or follow-up results of cases clearly reported?

Q9, was there clear reporting of the presenting site(s)/clinic(s) demographic information?

Q10, was statistical analysis appropriate?

the Newcastle–Ottawa Scale (NOS) for cohort study:

I, representatives of the exposed cohort;

II, selection of the non-exposed cohort;

III, ascertainment of exposure;

IV, demonstration that outcome of interest was present at the start of the study;

V, comparability of cohorts on the basis of the design or analysis;

VI, assessment of the outcome;

VII, was follow-up long enough for outcomes to occur?

VIII, adequacy of follow-up of cohorts.
